# A Quantitative Theory of Solid Tumor Growth, Metabolic Rate and Vascularization

**DOI:** 10.1371/journal.pone.0022973

**Published:** 2011-09-29

**Authors:** Alexander B. Herman, Van M. Savage, Geoffrey B. West

**Affiliations:** 1 Department of Radiology and Biomedical Imaging, University of California San Francisco, San Francisco, California, United States of America; 2 Department of Biomathematics, School of Medicine, University of California Los Angeles, Los Angeles, California, United States of America; 3 Santa Fe Institute, Santa Fe, New Mexico, United States of America; 4 Theoretical Division, T-8, Los Alamos National Laboratory, Los Alamos, New Mexico, United States of America; University of Sheffield, United Kingdom

## Abstract

The relationships between cellular, structural and dynamical properties of tumors have traditionally been studied separately. Here, we construct a quantitative, predictive theory of solid tumor growth, metabolic rate, vascularization and necrosis that integrates the relationships between these properties. To accomplish this, we develop a comprehensive theory that describes the interface and integration of the tumor vascular network and resource supply with the cardiovascular system of the host. Our theory enables a quantitative understanding of how cells, tissues, and vascular networks act together across multiple scales by building on recent theoretical advances in modeling both healthy vasculature and the detailed processes of angiogenesis and tumor growth. The theory explicitly relates tumor vascularization and growth to metabolic rate, and yields extensive predictions for tumor properties, including growth rates, metabolic rates, degree of necrosis, blood flow rates and vessel sizes. Besides these quantitative predictions, we explain how growth rates depend on capillary density and metabolic rate, and why similar tumors grow slower and occur less frequently in larger animals, shedding light on Peto's paradox. Various implications for potential therapeutic strategies and further research are discussed.

## Introduction

Tumor vasculature is a major target for cancer treatments. Vascularization links together the host tissue, from which vessels and blood are drawn, with the tumor mass and structure that is permeated by the vascular branching that supplies tumor cells with the necessary resources for its continued sustenance and growth. Consequently, a complete picture of angiogenesis must unite several disparate fields within tumor biology because it connects diverse properties of tumor cells, host vasculature, tumor metabolic rate and tumor growth. Understanding how these are integrated and interconnected is crucial for developing strategies for drug delivery and tumor treatment.

Tumors grow and are sustained by oxygen and resources delivered via the interface of the two coupled, but essentially autonomous, dynamical vascular networks of the host and the tumor. It is a major theoretical challenge to understand mechanistically the dynamics and geometry of this coupling. Part of the difficulty is that all tumor properties depend on *two* mass variables, that of the tumor and that of the host. Furthermore, tumors typically have significant amounts of necrotic tissue so the biologically active mass cannot simply be identified with the physical mass.

Over the past decade, new quantitative theories for the structure and hemodynamics of vascular networks in healthy mammalian circulatory systems have been developed to explain allometric scaling, with predictions that agree well with data [1], [2]. These models focus on the hierarchical, approximately self-similar properties of the branching network and can be used to calculate many physiological properties including blood flow rates in any vessel, vessel sizes and densities, and network structures and dynamics. The same framework has been successfully applied to the respiratory system and even to plants, which differ greatly in the physical structure and dynamics of their transport networks [1], [3]. Furthermore, it has been extended to quantitatively understand ontogenetic growth across mammals, birds and fish [1], and has inspired development of novel theory and collection of novel data [4]–[6]. As such, it is natural to extend this paradigm to address similar questions in tumor growth, structure and dynamics in order to develop an analogous quantitative theory for understanding many of their general growth and energetic properties. Combining recent empirical data on tumors with vascular modeling for healthy tissue provides an important method for analyzing tumor vasculature.

In parallel with these new models for healthy vasculature, models for tumor initiation and growth have emerged as a more central part of cancer research [7]–[9]. Most similar to our conceptual approach and goals are numerical simulations that describe how bulk properties of tumor cells, vascular networks and local host tissue control tumor growth [10]–[14]. These simulations are performed by parameterizing systems of partial differential equations and iterating them through time. Recent advances in hybrid continuos-discrete models allow detailed modeling over a wide range of spatial and temporal scales [10]–[14]. Within these models, the spatial growth of tumors is simulated within their microenvironment, and vascular networks are constructed by simulating the process of angiogenesis. Larger scales can be treated by decreasing the amount of information and level of resolution of cellular processes [13]. Such detailed modeling has produced a rich picture of the spatial features of tumor growth.

In this paper we construct a quantitative, predictive framework for understanding properties of tumor growth and vascularization that can be viewed as an application of allometric theory to tumor growth modeling. Specifically, we consider the relationship between the architecture of vascular networks and the metabolic and mitotic rates of individual cells. We focus on how key features of tumor growth and metabolism depend on architectural properties of tumor vasculature, such as vessel radii and lengths, and how growth and metabolic rates change dynamically with tumor size as well as with host size across species. We deliberately simplify our model by parameterizing it in terms of average, generic architectural properties of the tumor and host vascular systems and the energetics of creating, maintaining and replacing tumor cells. As such, our results are expressed in terms of relatively few, independently measurable, operationally defined parameters such as the average mass of a cell and the overall metabolic rate of the host. Consequently, our model largely coarse-grains over spatial heterogeneity, the physical interactions of the tumor cells with each other and the environment, and effects of competition between clonal lines of cancer cells. The benefit and power of our approach, however, is that we obtain analytical solutions that quantify and clarify the primary factors that affect tumor growth. These solutions provide a baseline for characterizing and understanding variation observed in empirical data and numerical simulations, and facilitate comparisons with healthy vasculature that lead to insights about optimization, altered growth rates and treatment strategies. Furthermore, our model provides a quantitative method for extrapolating parameters measured in mice and rats to be used in numerical simulations for tumor growth in humans. These numerical simulations, as mentioned above, can include more details about variation in space and across cell lineages.

We now detail our theoretical approach and data analyses. Geometric properties of tumor vasculature are measured using empirical data and compared against optimal predictions and known results for healthy vasculature. Using these results, we predict how tumor metabolic and growth rates depend on tumor and host masses. We also derive growth equations for the various phases of tumor development beginning with the diffusion driven pre-angiogenesis of very small tumors to the pulsatile driven angiogenesis of large, mature tumors. A major result of our theory, not addressed by other models and simulations, is the derivation of tumor metabolic rate and the recognition that the pulsatile nature of blood flow can play an important role in the structure, dynamics and growth of large tumors. Many specific quantitative predictions are made that compare well with data. One major prediction of our theory is to show how necrotic tissue necessarily arises from vascular inefficiencies, and to explicitly calculate how necrotic mass depends on tumor and host size.

## Materials and Methods

### Model

#### Derivation of tumor growth equation

The growth of a tumor is controlled by nutrient supply and demand. Solid tumors begin as avascular polyps dependent upon the diffusion of oxygen and nutrients across the tumor surface. Further growth depends on the recruitment and proliferation of blood vessels through angiogenesis and is fueled by metabolic resources in the host environment. In a process directly analogous to normal ontogenetic growth, incoming metabolites supply the tumor with energy and resources for creating new cells and maintaining existing ones [15]–[17]. Conservation of energy requires that the total metabolic rate of a tumor, 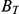, be apportioned between the power required for maintenance and that for mitosis:
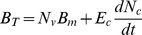
(1)where 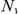 is the number of viable cells at time 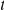 after growth begins, 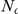 the total number of cells, 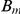 the power each cell requires for maintenance, and 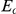 the energy to create a cell.

The rate of increase of the number of viable cells is the difference between the total rate of mitosis and the rate of cell death due to apoptosis and necrosis:
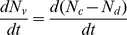
(2)where 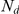 is the number of cells that have died by time 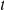. Even when the number of viable cells remains fixed (i.e., 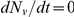), cells still die and are replaced by mitosis that requires tumor resources. Moreover, apart from natural causes, cell death also occurs due to nutrient deprivation because of inadequate or compressed vasculature. Thus, 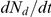 has contributions from different types of cell death, each potentially with its own functional form. Nevertheless, we can define the inverse lifetime of an average cell, 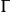, by
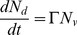
(3)Eqs. (2) and (3) closely resemble ones used in Macklin et al 2009, but they invoke a dimensionless time by dividing by a fixed inverse mitosis rate [10]. In contrast, we allow the inverse cell lifetime to vary with tumor type, as determined from fits to tumor growth data described below.

Combining Eqs. (2) and (3) we can re-express the growth equation, Eq. (1), purely in terms of viable cells:
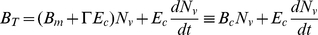
(4a)where 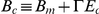 is the average cellular metabolic power required for maintenance and replacement. Note that the specific metabolic rate of the tumor (i.e., per tumor cell) is proportional to the oxygen concentration, such that 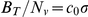, where 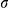 is the oxygen concentration and 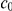 is a well-known conversion factor for converting the volume flow rate of oxygen into power. Eq. (4a) can then be expressed as
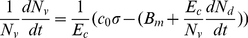
(4b)showing explicitly how tumor growth depends on oxygen concentration, easing the comparison with other models. In Macklin et al 2009, for instance, the net proliferation rate (equal to the volume rate of change) 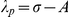, where 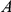 is the rate of apoptosis. Our equation for proliferation is similar, but is derived mechanistically, from the energetics of the whole tumor, and thus includes two additional terms not explicitly accounted for by Macklin et al: 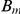 reflecting the oxygen used for maintaining existing cells and 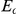 capturing the cost of cell creation.

Since the viable tumor mass, 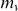, is the product of the total number of viable cells and the average mass of a cell, 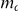, we have 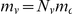 leading to
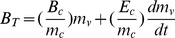
(5)This first-order differential equation, representing conservation of energy, explicitly links properties of tumor cells (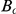, 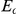, and 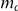) with properties of the whole tumor (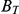 and 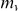). Consequently, it provides a simple, but powerful, way to integrate important features and results from different areas of cancer research. Solving this equation to determine tumor growth requires knowledge of how tumor metabolic rate, 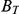, depends on its viable mass, 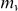, to which we now turn.

#### Model for tumor vascular system and the prediction of metabolic rate

Tumor metabolic rate, 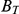, is proportional to the sum of the rates of cellular fermentation and aerobic respiration. For avascular tumors, 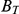 depends on the diffusion rate of nutrients and oxygen from the surrounding environment [18]. For vascular tumors, 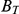 is proportional to the total blood volume flow rate to the tumor, 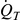, consistent with observations that glucose and oxygen consumption rates vary linearly with blood flow rate [19]. The dependence of 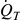 on 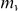 and host mass, 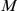, is determined by the structure, dynamics and interaction of the tumor and host vasculatures. Here, we develop a complete analytical model of tumor vascular networks applicable throughout different phases of development by deriving the allometric scaling of tumor rates and times with host body size and capillary density. Although the importance of the vascular interface between the tumor and the host has been previously recognized, our work is a novel attempt to mechanistically model its role in tumor growth [10]–[12], [20].

Mounting evidence suggests that some tumor vascular networks exhibit fractal-like properties similar to those of the circulatory system [21]–[23]. To analyze tumor vasculature, we borrow from an idealized framework that has proven successful for quantitatively understanding the circulatory system. This framework assumes that in healthy tissue the vasculature is space-filling, minimizes energy loss and has invariant terminal units (capillaries) [1]. We compare these optimal networks with measures of tumor vasculature, while retaining the assumption of invariant capillaries.

**Figure 1:**
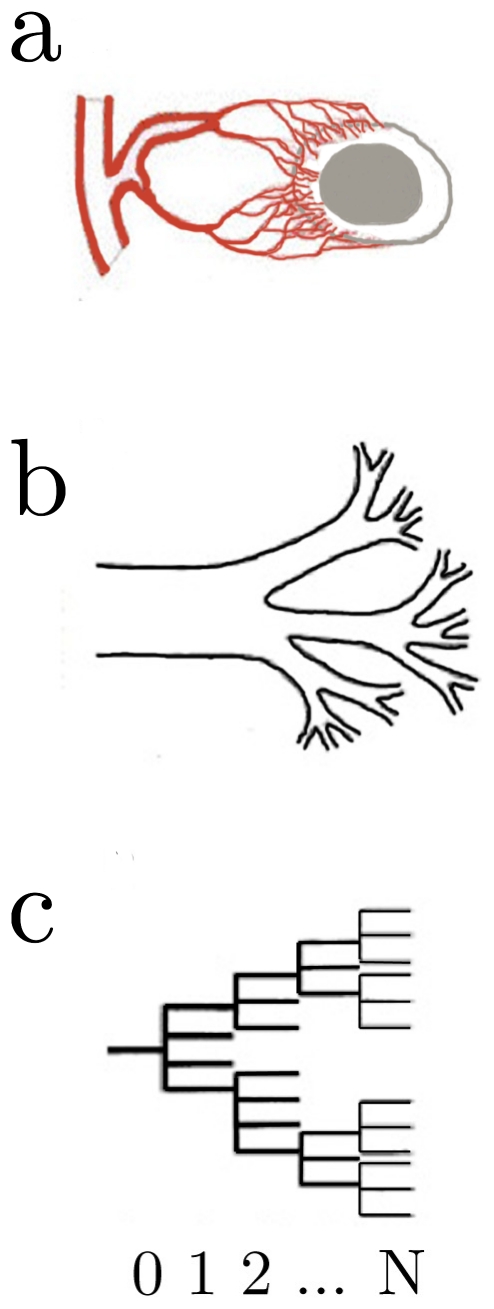
Schematic of tumor growth model. (a) Vascularized tumor supplied by blood siphoned from host vasculature. White area represents viable tissue, while grey represents necrotic core. (b) Schematic of vascular network composed of tubes. (c) Topological model of tumor and host network beginning with feeding vessel (k = 0) and terminating at the capillary level (k = N).

To facilitate comparisons between healthy and tumor vasculature, we introduce scaling ratios for radii and lengths of vessels across levels, 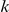, of the network. We treat all branches at the same level, 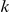, as having similar properties and assume a constant branching ratio, 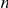
–the effective number of daughter vessels for each mother vessel [1]. Following West et al 1997 and Gevertz et al 2006, we model blood vessels as cylinders, similar to the Krogh model [1], [11]. The capillaries define the lowest level 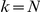 while the largest vessels feeding the tumor define 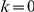 (Fig. 1). We introduce scale factors for the ratio of daughter to mother vessel radii:
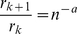
(6)and similarly for daughter to mother vessel lengths:
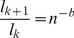
(7)The exponents, 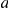 and 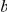, can be used as quantitative diagnostics for comparison with healthy tissue, where theory predicts and data support 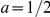 for large vessels and 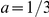 for small vessels (from energy minimization) and 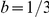 for all vessels (from space filling) [1]. Deviations from these values indicate the degree to which optimization and space-filling are violated during tumor growth.

**Figure 2:**
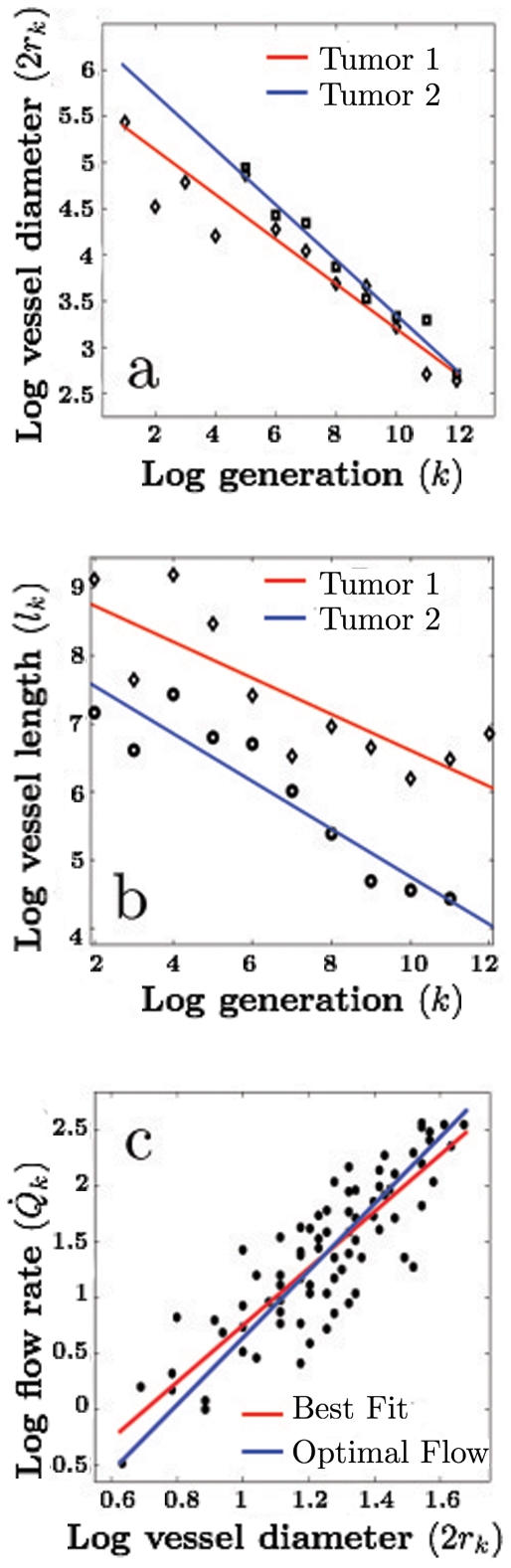
Properties of tumor vascular networks. (a) Plot of log 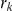 versus 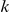. The absolute value of the slope represents the exponent 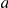, defined as the ratio of radii between consecutive levels: 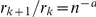. Tumor 1: Mammary Carcinoma 1 (red line/squares): 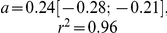. Tumor 2: Colorectal Carcinoma (blue line/diamonds): 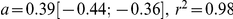. (b) Same as (a) but for the ratio of lengths between consecutive levels: 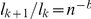. Tumor 1: Mammary Carcinoma (red line/diamonds): 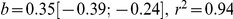. Colorectal Carcinoma (blue line/circles): 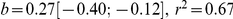. (c) Plot of the logarithm of flow rate versus logarithm of vessel diameter, showing the predicted idealized cubic law (blue line 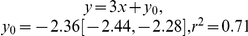) and the best linear fit (red line 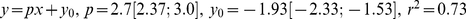). Data from [21] and [57].

For healthy tissue, 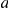 and 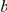 are approximately independent of 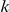, indicating that the network has a fractal-like structure, as observed. To determine if tumor vascular networks have similar geometric structure, we observe that for vessel radii, 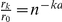, where 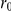 is the largest vessel in the hierarchy, and taking the log of both sides and rearranging yields 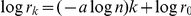, and similarly for vessel lengths 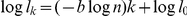, so plotting 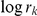 and 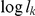 versus 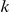 should yield straight lines whose slopes are 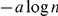 and 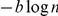, respectively, if 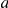 and 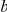 are constant. Figs. 2a, 2b show data from various tumors, indicating that tumor vasculature does indeed exhibit approximately fractal behavior, in agreement with other studies [22], [24].

The metabolic rate of the tumor, determined by oxygen and nutrient availability, depends on its capillary density, which is controlled by the scaling factors 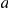 and 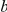. In File S1 we derive the relationship between the metabolic rate, tumor size and vascular architecture:
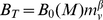
(8)where 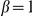 if 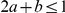, but 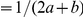 otherwise, and 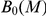 is a normalization factor that depends on the host mass, 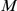. For healthy tissue, where capillary density is controlled by large-vessel scaling, this gives 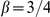, in agreement with data (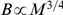) for large mammals [25]. For tumors too small to support significant pulsatile flow, or whose host supply vessels are likewise too small, theory predicts 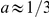. So, if their vasculature is space-filling, 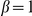 and their metabolic rate scales linearly: 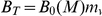 [1].

As tumor vasculature becomes increasingly inefficient and/or attaches to host supply vessels sufficiently large to deliver pulsatile flow, 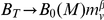 with 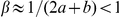, ultimately decreasing towards 3/4, similar to whole body scaling [2]. Indeed, changes in this scaling exponent have been observed by Guiot *et al* who determined 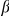 by matching the general form of our growth equation to empirical data and speculated these changes were tied to the fractal dimension of the vasculature [26]. To the extent that a tumor network deviates from optimized space-filling architecture, it will exhibit a secondary reduction in blood flow leading to hypoxia and necrosis determined by the value of 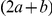. This is related to the development of avascular areas due to the irregular space-filling properties observed in tumor vasculature growth [27]. During growth, tumor blood flow shows a reduction in mass-specific blood flow rate resulting in increased necrosis [28]–[31], consistent with this theory.

#### Model for interface of tumor and host vascular systems

We now examine how the host vasculature interfaces with the tumor to determine the dependence on host mass, 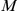, and the degree of necrosis. While previous work has suggested relationships between host size, tumor growth and metabolism [17], [32], [33], little underlying theory has been developed, especially concerning the role of vascularization. Many tumor growth models that consider vasculature track how blood flows and oxygen diffuses to the tumor from a host parent vessel. Our model goes significantly further by considering the entire flow through and structure of both networks, thereby showing how the blood supply from the host changes with host body size, the size of the parent supply vessels, and the nature of the blood flow (whether it is laminar or pulsatile). Almost all previous investigations presume a simple laminar flow given effectively by the classic Poisseuille formula with time-invariant blood pressure. Our model includes the critical role of pulsatile flow for large tumors and considers its consequences for tumor growth and metabolism, leading to novel and substantially different predictions than models based purely on laminar flow.

For very small tumors, resources for metabolism are supplied by host capillaries that have been incorporated and displaced from the surrounding tissue. In File S1, we derive how, in this case, capillary density and tumor metabolic rate depend on tumor and host size, yielding
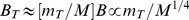
(9)where 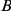 is the metabolic rate of the host. Thus, the tumor metabolic rate, 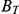 increases linearly with *total* tumor mass, 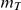, but decreases with host mass as 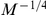. However, from our network analysis for small tumors, we had 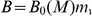. Equating these gives 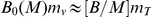, 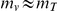 and 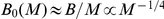. This predicts that, initially, little necrotic tissue develops and that tumors begin growth approximately exponentially as 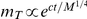, where 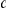 is a constant depending on tumor type and microenvironment. Consequently, similar tumors grow systematically slower in larger animals due to their lower mass-specific metabolic rate, as noted for humans [17]. More generally, growth rates are predicted to depend on host capillary density and metabolic rate in the tissue surrounding the tumor, in agreement with previous models [10]–[12].

As tumors grow further, an anastomotic network forms from the local host vasculature, usually at the arteriole level, eventually either penetrating the tumor surface or becoming incorporated into it [34]. Further growth leads to and is stimulated by recruitment of increasingly larger supply vessels. The host tissue from which the tumor draws blood is effectively a shell whose thickness depends on the distance 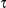 that tumor angiogenic factors penetrate into the surrounding host tissue. This distance depends on production and consumption rates of angiogenic factors as they diffuse into the local tissue environment [10]–[12]. Rather than simulating this process numerically using a detailed reaction-diffusion model as others have done, we employ a classic diffusion equation argument and take the diffusion distance, 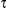, to vary inversely with endothelial cell density. This density, in turn, is determined by the surface area of host vasculature (see also Macklin, Chaplain and Gevertz et al. [10]–[12]). In File S1, we derive relationships between tumor blood flow rate, 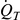, diffusion distance 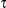, endothelial cell density, and tumor and host size, yielding
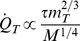
(10)From the analysis of large tumors given above, we had 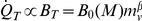 with 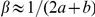. Equating this with Eq. (10) then gives
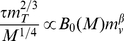
(11)Furthermore, from Eq. (6) in File S1, 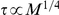, so Eq. (11) implies
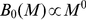
(12)That is, 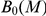 is independent of 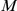, and
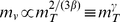
(13)where 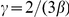. Consequently, when the tumor develops its own vascular system, this predicts the non-intuitive result that the metabolic rate of tumors of similar size no longer depends on host size. Thus, as tumors grow, the effects of host vessel density and shell size compensate each other, dampening the dependence of tumor metabolic rate on the host properties. Intriguingly, this finding, Eq. (13), also predicts that viable tumor mass increases at a slower rate than total tumor mass, necessarily implying the development of necrotic regions within the tumor.

In summary, when tumors are small, their metabolic rate is limited by host capillary density, which decreases with host size as 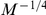, whereas when tumors are large, this effect is counteracted by the tumor's access to more and larger host vessels. This result embodies basic constraints arising from the interface of the host and tumor vasculature. It has important consequences for determining the dynamics of various properties, such as the degree of necrosis, as we now see by examining two limiting phases of growth.

#### Tumor growth dynamics

Having determined how tumor metabolic rate scales with viable tumor mass, we discuss its consequences for tumor growth dynamics by returning to Eq. (5). Using 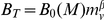 from Eq. (8), the conservation of energy equation can be rearranged into a growth equation:
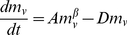
(14)where the parameters 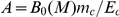 and 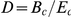 embody generic properties of tumor cells whereas properties of the host physiology are reflected in 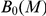.

As articulated above, these coefficients and the exponent 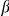 potentially change (in a calculable way) with time as the tumor evolves through different phases of development. For simplicity, as well as for illustrative purposes, we consider here the exponential and sigmoidal phases of growth as distinct regimes where the parameters remain fixed. The general solution to Eq. (14) is
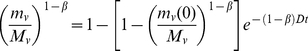
(15)where 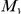 is the asymptotic viable mass of the tumor, as computed below in Eq. (18), and 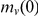 is the initial malignant mass. Since 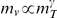, these equations can be transformed into *identical* equations for the total tumor mass, 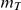, but with different values of the parameters. This leads to the following important result: the viable and total tumor masses both satisfy growth equations of the same form as healthy tissue but with different exponents and coefficients.

In the initial stages of tumor growth when delivery of resources is via diffusion from nearby capillaries, the total and viable tumor masses are linearly related (Eq. (9)). Furthermore, 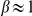 (see File S1). Substituting these into Eq. (15) leads to exponential growth for both the total and viable tumor masses:
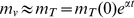
(16)with 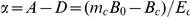, determined by both cellular and host properties.

In the early non-pulsatile regime, our model still predicts that 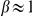, so from Eq. (15) exponential growth continues for the viable mass. However, in this regime, viable and total tumor mass are now *non*-linearly related, according to Eq. (13), so the total tumor mass grows exponentially at a faster rate than the viable mass, leading to a monotonically increasing proportion of necrotic tissue. This can be expressed as
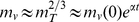
(17)So, for small tumors that can neither support nor are supplied by pulsatile blood flow, this predicts 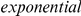 growth for the 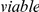 tumor mass.

Note that, if 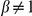, the initial growth for small times is 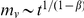. However, if 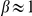, this is almost indistinguishable from an exponential, so most tumors are expected to begin growth approximately exponentially. As long as large host supply vessels that support pulsatile flow do not develop, this behavior will continue until other physical constraints become limiting or the vascular supply is exhausted or interrupted. When angiogenesis begins and tumor vasculature develops, resource supply does not match demand because 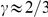, and the relative rate of increase of 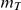 is a factor 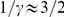 greater than for 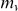. Thus, the proportion of viable tissue, 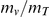, decreases exponentially quickly.

In later stages we showed above that 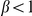; from Eq. (15) this leads to classic sigmoidal growth with the viable mass reaching a fixed asymptotic value given by
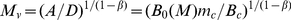
(18)This is reached when 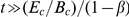; 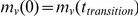 is the corresponding mass at the time of transition from exponential growth to the initiation of the sigmoidal phase being considered. In the later vascular phase dominated by pulsatile flow, 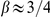 and 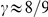, so Eq. (15) leads to sigmoidal growth with 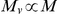, with viable mass directly proportional to host mass. The asymptotic mass of the whole tumor is 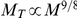, suggesting proportionately larger tumor sizes in larger animals, consistent with the limited available data.

## Results

Many tumor vascular properties can be derived and compared with experiment, as well as with observed data for healthy vasculature. For example, total vascular surface area, 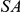, is predicted to scale linearly with the total number of capillaries, and therefore linearly with the total blood volume flow rate, giving 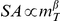, consistent with experimental observations [31], [35]. Furthermore, in an optimized network, the blood volume flow rate in a vessel at level 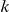 is predicted to follow a cubic law: 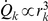. Figure 2c depicts measurements from carcinomas that are consistent with this prediction, although the greater variance suggests tumor vasculature is less well-formed than healthy vasculature. In addition, the total hydrodynamic resistance of the network, 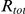, is predicted to vary inversely with the total number of capillaries, and hence inversely with total blood flow rate giving
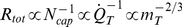
(19)This equation can be rearranged to predict 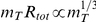 (see File S1). Data for this relationship gives an exponent of 0.36, in good agreement with the prediction of 1/3 [36].

**Figure 3:**
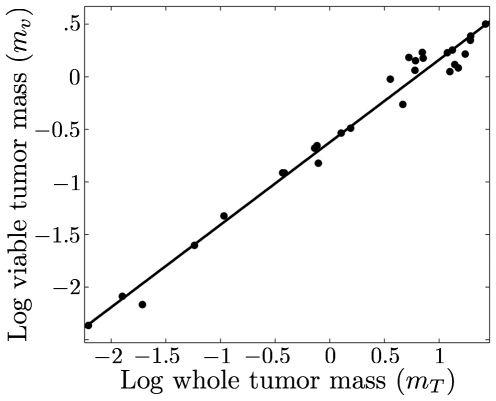
Scaling of tumor viable mass, 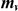
 **, as a function of the total tumor mass,** 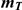 **.** Theory predicts that 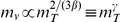. In this case, 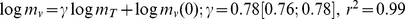, so that for these tumors 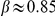, implying a high blood-flow/metabolic rate. However, since these data are drawn from multiple tumors, it represents an estimate. Data from [58].

The predictions for the increase in size of the necrotic core and viable mass, (Eq. (17)), are also consistent with both available data (Fig. 3), and with numerical simulations [10]. Indeed, tumors develop significant amounts of necrotic tissue both in their core and in regions throughout the tumor as a result of insufficient angiogenesis, vascular collapse and an underdeveloped lymphatic system for the clearance of dead cells [37], [38]. Finally, since the rate constant for growth in Eq. (15) is 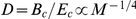, similar to that in normal ontogeny, typical tumor time-scales such as doubling time, cell cycle time, and time to death are all predicted to scale with host mass as 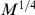, consistent with the limited available data (doubling time exponent 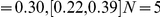, cell cycle time exponent 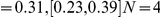) [39], [40].

**Figure 4:**
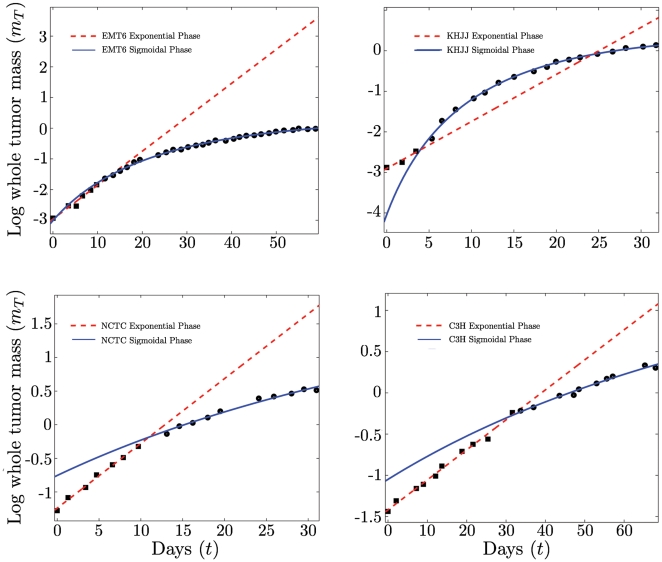
Fits of the growth equation to empirical data for tumor growth. Exponential, from Eq. (16), and sigmoidal, from Eq. (15), regimes of the growth equation, where 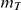 is the tumor mass at time 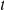, 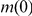 is the initial mass, 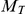 is the asymptotic mass, 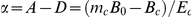 is the rate of exponential growth, 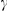 is the viable mass scaling exponent, and 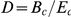 is a characteristic time constant for tumor cells that is given by the ratio of the metabolic rate of a tumor cell to the energy to create a tumor cell. Fits to several types of tumors implanted in mice and rats yield the parameter values with corresponding confidence intervals of: (a) EMT6 exponential: 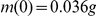, 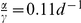; EMT6 sigmoidal: 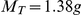, 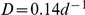. (b) KHJJ exponential: 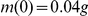, 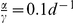; KHJJ sigmoidal: 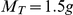, 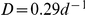. (c) NCTC2472 exponential: 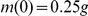, 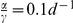; NCTC2472 sigmoidal: 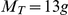, 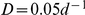. (d) C3H exponential: 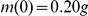, 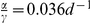; C3H sigmoidal: 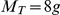; 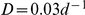. Using 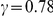 based on the fit from Fig. 3, we compute that for the tumor types in panels (a)–(d), 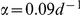, 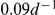, 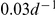, and 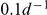 respectively, which is remarkably consistent given the amount of error in the data. Data from [40].

To investigate the accuracy of our growth models and the transition between different growth regions, we fitted Eq. (15) to tumor growth data from the literature, as shown in Fig. 4. Data are insufficient to distinguish statistically between combined exponential and sigmoidal fits to different growth phases versus a single sigmoidal fit to the entire dataset. Nevertheless, our fits are in good agreement with empirical data and show the clear transition from early exponential growth to late sigmoidal growth.

## Discussion

Our model mechanistically and quantitatively connects properties of tumor cells to multiple properties of the whole tumor. Although previous models have reproduced the sigmoidal behavior of tumor growth, our derivation of the growth equations is novel because it allows the extraction of observable, metabolic quantities from our data fits that can be used to compare metabolic properties of different tumor lines with each other and with normal tissue [13], [41]. It is notable that the confidence intervals for parameter values estimated from our fits to sigmoidal growth overlap with the range observed for normal growth. This finding suggests that tumor cells retain the gross metabolic features of normal cells, which is consistent with earlier work by Skehan who used a large number of statistical models to conclude that the gross properties of tumor growth are almost indistinguishable from normal tissue growth [42]. The model also allows us to predict that whole tumors will grow faster if their constituent cells have low 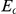, high 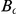 or both. This is consistent with the observation that many aggressive tumors are inchoate and poorly differentiated, and that their cells exhibit elevated metabolic rates. Similarly, relatively benign tumors are expected to show higher levels of differentiation and lower mass-specific rates of energy use. If 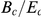 is not significantly different from its value for healthy cells, this suggests that at late stages such tumors grow at relative rates comparable to that of healthy organs during ontogeny.

Our framework makes several predictions that relate to the diagnosis and treatment of solid tumors. First, it clearly distinguishes between different growth regimes: 1. Pre-angiogenesis/diffusion regime, 2. Early angiogenesis/smooth, laminar non-pulsatile blood flow regime, and 3. Late angiogenesis/pulsatile flow. In regimes 1 and 2, we predict that growth rates are systematically much faster in smaller mammals (Eq. (9)), whereas in regime 3, growth rates are independent of host body size (Eq. (12)). This suggests that early detection of tumors is even more critical than currently recognized because that is the regime during which tumors in humans exhibit proportionately slower growth rates than in smaller mammals. Moreover, these results also suggest that different treatments should be developed for, and tailored to, these different growth regimes.

Our results have potentially important consequences for scaling up experimental findings from mice to humans. One testable prediction of our model, confirmed by preliminary evidence, is that human to mouse tumor xenografts will grow at a rate similar to endogenous mouse tumors, as opposed to human tumors [39], [40]. Differing predictions for different growth regimes derived above suggest that treatment and drug dosages obtained from mice studies must be properly scaled up and applied to humans only after careful consideration of both the mouse's and patient's tumor growth regimes.

Resolving questions about cancer incidence rates in different species, and thus Peto's paradox, may also be possible by considering scaling consequences as a function of body mass and metabolic rate [43]–[45]. Tumors must develop a number of specific mutations along a single cell lineage to become malignant, probably in a specific order [7], [46]. Although the total number of cells in the body increases almost linearly with body size, the number of cell-generations increases only logarithmically with body size [47]. If most cancer-causing events occur during cell division, the whole-body probability of developing a lineage of mutations is then proportional to the product of the number of generations of cell division and the probability of mutation per cell generation. The power density driving biochemical reactions within a cell scales as 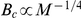, and if these biochemical reaction rates drive mutation rates, for instance through the production of reactive oxygen species, then overall cancer incidence rates would scale as 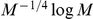, which becomes dominated by the 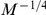 term after the first couple orders of magnitude [48]. Indeed, this leads to the sensible prediction that cancer incidence rates scale inversely to maximum lifespan, which scales approximately as body size to the quarter power (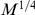) [49]. Thus, smaller mammals are systematically expected to have a greater incidence rate of a given tumor than larger mammals.

In addition, our theory sheds light on how a large number of rates and time-scales considered in numerical models, such as tumor angiogenic factor production, cell-cycle time, rate of apoptosis and necrosis, and oxygen concentration, should be adjusted with tumor and host size. For some of these parameters, such as tumor angiogenic factor production rate 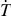, we can immediately predict how they scale with host and tumor size: early on for small tumors, as 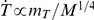 whereas, later for large tumors, as 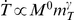.

Although we made simplifying assumptions to reflect existing quantitative knowledge of tumor kinetics, the framework can be straightforwardly extended to capture more complex details of tumor growth. For example, tumor metabolic scaling will vary depending on how the architecture of specific tumor vascular networks deviate from optimality. Therefore, no single power law will exactly describe the host size dependence across all tumors.

Our model does not explicitly treat spatial features of tumor growth. Moreover, many parameters that are explicit in other models are implicit components of our model, including 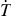, blood-tissue oxygen transfer rates, the oxygen diffusion coefficient and the oxygen concentration threshold for quiescence and necrosis [10]. Currently, the cellular energetic parameters 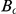, 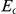 and 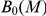, and the scaling exponents, subsume the average effects of spatial processes such as cell motility. More detailed models would allow the decomposition of these parameters into underlying microscopic processes constraining tumor growth that are considered explicitly in numerical approaches. For example, a reaction-diffusion model for angiogenic factor gradients and host vessel recruitment, or a lattice-model of cellular motility and cell-cell adhesion, would allow for more fine-grained simulations of spatially resolved tumor vasculature and growth co-development [10], [12]–[14], [50]. Cell motility could be expressed in terms of the energy used by cells to move and thus be connected to the cell's energy budget. Because of energetic constraints, the cell velocity or number of motile cells in a spatial-version of our model would thus be constrained by the blood supply and hence tumor and host size.

Furthermore, many models of tumor growth include three distinct layers: proliferating, quiescent and necrotic [10], [13], [14], [50]. In our model, we combine the proliferating and quiescent layers. Modeling proliferating and quiescent cells as separate layers with a transition region would involve the expansion of our theory to capture the effect of oxygen concentration on cell state, as influenced by tumor and host size. Because oxygen concentration in the tumor is the main determinant of the balance between the number of proliferating and quiescent cells, we expect the number of proliferative cells to decline across host species with increasing host mass as 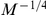 during the early growth stage and then scale only with tumor size as the tumor grows larger. Hence, studies in small mammals (e.g., mice) of therapies such as chemotherapy and radiation, which are targeted at actively dividing cells in relatively small tumors, may result in an effect larger than what should be expected in humans.

We assumed that the inverse cellular lifetime, 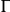, is independent of time or tumor size, consistent with the constant mitotic rate assumed in other models such as Macklin et al 2009. A more detailed model could account for dependencies on viable mass due to factors such as heterogeneous spatial distribution of blood flow and interstitial fluid pressure [51]. As long as these effects are small, our theory gives a leading-order description of tumor necrosis, although it cannot predict its spatial distribution. Additionally, while the cellular energetics parameters 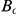 and 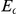 may vary during tumor growth due to a changing environment and the emergence of new cell phenotypes, we have suppressed a sum over cell types and clonal populations and have made the simplification that tumors are composed of average cells. Extending our model to include growth parameters that evolve with emerging clonal populations may prove useful in understanding how drug dosages in chemotherapy could be dynamically adjusted [52]. Accurate *in vivo* measurements of tumor metabolic and mitotic rates, increasingly feasible with advances in imaging technology, would also allow us to provide more precise predictions for the behavior of growing tumors, and may also be useful in describing effects of the approach of normalizing and then destroying tumor vasculature [53]. More exact measurements of tumor growth rates would allow for tighter bounds on the values of 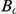 and 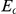 derived from growth fits, and conversely, more detailed and accurate measurements of tumor cellular metabolic properties would allow for the quantitative prediction of tumor growth.

To summarize: we have presented a general quantitative framework that captures many of the essential features of tumor vascularization and growth, and how these are influenced by the host organism. We derived predictions for many rates. times and sizes of both solid tumors and their vascular networks as they grow and interconnect. In addition, we predict how many of these properties depend on host body size, thus laying groundwork for resolving the long-debated issue of how cancer incidence rates scale from mice to humans [54]. Similarly, these results may help us understand how to scale results from experiments on model organisms up to humans, possibly through scaled parameterizations of numerical models, and also how drug dosages are affected by tumor metabolic rate, vascularization and growth stage [55], [56]. By focusing on metabolic rate, our integrative model allows for quantitative comparisons between tissue and cellular level growth for tumors, as well as comparisons of these quantities among different types of normal tissue and solid tumors.

## Supporting Information

File S1 (PDF)
