# Supplementary material for: A Quantitative Theory of Solid Tumor Growth, Metabolic Rate and Vascularization
Source: PLoS One. 2011 Sep 29;6(9):e22973. doi: 10.1371/journal.pone.0022973 (PMC3182997; doi:10.1371/journal.pone.0022973)
Supplement: File S1 — (PDF) [file pone.0022973.s001.pdf]

Supporting Information File S1

Alexander B Herman[1,3,4,*], Van M Savage[2,3,4], Geoffrey B West[3,4]

1 Department of Radiology & Biomedical Imaging/Medical Scientist Training Program, Univeristy of California, San Francisco, San Francisco, CA, USA

2 Department of Biomathematics, University of California, Los Angeles, Medical Center, Los Angeles, CA, USA

3 Santa Fe Institute, Santa Fe, NM, USA

4 Theoretical Division, Los Alamos National Laboratory, Los Alamos, NM, USA

∗ **E-mail: alexander.herman@ucsf.edu**

Derivation of metabolic scaling

The total volume flow rate of blood to the tumor, $\dot{Q}$, is the product of the total number of supply vessels, N_0, and the volume flow rate in each of them, $\dot{Q}_0$. By fluid conservation we have $\dot{Q} = N_0\dot{Q}_0 = N_{cap}\dot{Q}_{cap}$, where N_{cap} is the total number of capillaries, each with flow rate $\dot{Q}_{cap}$. The total blood flow rate for the tumor is thus directly proportional to the total number of capillaries, $\dot{Q} \propto N_{cap}$. The total blood volume in the network, summed over all vessels, is $V_b = \sum_{k=0}^{N} N_k V_k = \sum_{k=0}^{N} \pi n^k r_k^2 l_k$, where k is the generation number, N is the number of generations from feeding vessel to capillary, and V_k is the volume of a vessel at level k. A further prediction of energy minimization is that V_b scales linearly with (viable) mass, in agreement with data for some tumors ($V_b \propto m_v$) and for the whole body ($V_b \propto M$), although this relationship may change for some tumors. We can then relate the total blood volume V_b, to the total number of capillaries, $N_{cap} = n^N$ [22]. When the number of vascular levels $N >> 1$, as expected for most tumors, the result depends critically on the magnitude of the scaling exponents, a and b: (i) when $2a + b < 1$, blood volume scales linearly with capillary number, $V_b \approx [N_{cap}/(1 - n^{(2a+b-1)})]V_{cap}$; (ii). however, when the opposite condition holds, $2a + b > 1$, V_b scales super-linearly with capillary number, $V_b \approx [N_{cap}^{(2a+b)}/(1 - n^{(1-2a-b)})]V_{cap}$. Consequently, because $V_b \propto m_v$, the number of capillaries scales as a power law with the viable tumor mass. When $2a + b < 1$, the dependence is linear, N_{cap}/m_v, whereas,

when $2a + b > 1$, it is non- linear: $V_b N_{cap} \propto m_v^{1/(2a+b)}$. It immediately follows that $N_{cap} \propto m_v^{\beta}$, where $\beta = 1$ if $2a + b \leq 1$, but $= 1/(2a + b)$ otherwise. Total blood flow rate, $\dot{Q} \propto N_{cap}$, is therefore predicted to exhibit power-law scaling with *viable* tumor size: $\dot{Q} \propto m_v^{\beta}$, consistent with a large amount of data and past conclusions. Finally, since the rate of supply of nutrients and oxygen is directly proportional to blood flow rate, we have for the metabolic rate of the tumor

$$B_T = B_0(M) m_v^{\beta} \tag{1}$$

with $B_0(M)$ a normalization factor that, in principle, depends on host mass, M.

Network Resistance

The resistance of the network can be found by summing over the resistances of each level in the network. The viscous resistance to blood undergoing steady, laminar flow is given by the Poiseuille formula $R_k = 8\mu l_k/\pi r_k^4$, where R_k is the resistance of a vessel at level k in the network. Setting $\delta = r_{k+1}/r_k$ and $\lambda = l_{k-1}/l_k$, and ignoring small effects such as turbulence and non-linearities at junctions, the total resistance for the tumor is given by

$$R_{tot} = \sum_{k=0}^{N} \frac{R_k}{N_k} = \sum_{k=0}^{N} \frac{8\mu l_k}{\pi r_k^4 n^k} = \frac{[1 - \left(n\delta^4/\lambda\right)^{N+1}] R_{cap}}{\left(1 - n\delta^4/\lambda\right) n^N} \tag{2}$$

where μ is the blood viscosity. Now, if $n\delta^4/\lambda < 1$ and $N \gg 1$, a good approximation is $R_{tot} = R_{cap}/\left(1 - n\delta^4/\lambda\right) N_{cap}$. Assuming that R_{cap} is invariant, $R_{tot} \propto N_{cap}^{-1}$, with the smallest vessels in the network dominating the resistance. Since $n\delta^4/\lambda < 1$ for the malignant tissues described in this paper, the tumor resistance is inversely proportional to the number of capillaries, and hence the total blood flow rate, or $R_{tot} \propto \dot{Q}_0^{-1} \propto m_T^{-\beta}$. This predicts that $R_{tot} \propto m_T^{-2/3}$, consistent with results from [1], where the resistance to flow in tumors of various weights was measured as the slope at the point where the flow rate $\dot{Q}_T$ became linearly proportional to pressure. Without knowing the actual perfusion pressure and flow rate *in vivo*, this is a good approximation to the resistance of the tumor vascular network.

Metabolic rate of small tumors

Very small tumors are supplied by the diffusion of nutrients from nearby host capillaries so that the supply to the tumor is proportional to the product of the number of host capillaries within the diffusion distance from the tumor, N_T^{cap}, and the flow rate through each host capillary, Q^{cap}. In this initial stage we assume that all host capillaries displaced by the tumor volume, V_T , act as supply sources because they are adjacent to the tumor and within a diffusion distance of its surface. The total blood flow rate to the tumor is then given by

$$\dot{Q}_T \approx N_T^{cap} Q_{cap} \approx [\rho^{cap} V_T]\dot{Q}^{cap} = \frac{V_T}{V}[N^{cap}\dot{Q}^{cap}] \tag{3}$$

where $\rho^{cap}(\approx N^{cap}/V)$ is the average capillary density of the host and N^{cap} their total number in the whole body, whose total volume is $V \propto M$. The whole body flow rate is $\dot{Q}_0 = N^{cap}\dot{Q}^{cap} \propto M^{3/4}$, and, since tissue density is approximately constant (i.e., $V \propto M$ and $V_T \propto m_T$), this gives $\dot{Q}_T \approx [m_T/M]\dot{Q}_0 \propto m_T/M^{1/4}$. This then predicts that the tumor metabolic rate is

$$B_T \approx \frac{m_T}{M} B \propto \frac{m_T}{M^{1/4}} \tag{4}$$

Thus, for very small tumors, B_T is predicted to increase linearly with *total* tumor mass, m_T, but decrease with host mass as $M^{-1/4}$.

Recruitment of vessels from host

The host tissue from which the tumor draws blood is effectively a shell surrounding the tumor with thickness determined by the diffusion distance τ, and volume τS_T, where S_T is the tumor surface area. The diffusion distance, τ , which depends on production and consumption rates of angiogenic factors, is assumed to vary inversely with endothelial cell density. The endothelial cell density in the tissue shell is determined by the density of the surface area of host vasculature, $\sum_k 2\pi N_k r_k l_k/V$. Since capillaries have constant surface area and often dominate this scaling, the endothelial cell density scales linearly with capillary density, ρ_{cap}. For large tumors, this gives

$$\tau \propto \rho_{cap}^{-1} \propto M^{1/4} \tag{5}$$

This result is consistent with the idea that tumors attach to the host vasculature several levels above the capillaries and that tumors are able to attach to successively larger vessels in larger hosts.

If the host supply vessels are recruited from the $k = L$th level of the host hierarchy, then the total number of such supply vessels in the shell of thickness τ is $\sim \rho_L S_T \tau$, where $\rho_L \approx N_L/V$ is their average density and N_L their total number in the whole body. If all these vessels are recruited by the tumor, they contribute

$$\dot{Q} \approx [\rho_L S_T \tau] \dot{Q}_L \tag{6}$$

to its total blood supply rate, where $\dot{Q}_L$ is the flow rate in an Lth level vessel. Assuming Euclidean scaling, $S_T \tau \propto V_T^{2/3} \propto m_T^{2/3}$ and using fluid conservation $\dot{Q}_0 = N_L \dot{Q}_L$ gives

$$\dot{Q} \propto \frac{\tau m_T^{2/3}}{M} [N_L \dot{Q}_L] \propto \frac{\tau m_T^{2/3}}{M^{1/4}} \tag{7}$$

Development of necrotic core during angiogenesis: Two regimes

An important consequence of our theory is that it predicts the existence of necrotic tissue. Indeed, it allows a determination of the degree of necrosis as a function of tumor size, as we now show by considering two limiting phases of growth.

i) The early phase where supply vessels are host capillaries and arterioles. In this case $\tau \propto M^0$ is approximately invariant and $\beta \approx 1$ so $\dot{Q} \propto B_T = B_0(M) m_v$ with $B_0 \propto M^{-1/4}$. This behavior is similar to the initial diffusion phase where similar scaling is manifested. However, the viable mass, m_v, no longer scales linearly with the total tumor mass, m_T, but now scales non-linearly: $m_v \propto m_T^{2/3}$, thereby predicting an increasingly larger proportion of necrotic tissue as the tumor grows, namely,

$$\frac{m_v}{m_T} \propto m_T^{-1/3} \tag{8}$$

ii) The later phase when the tumor has access to more and larger vessels, and tumor vascular inefficiencies become increasingly important. In this case, $\tau \propto M^{1/4}$ and $B_0(M)$ is approximately independent of M. Moreover, we find that as the tumor grows, β evolves from 1 towards 3/4 implying that γ changes from 2/3 to 8/9 so that ultimately

$$\frac{m_v}{m_T} \propto m_T^{-1/9} \tag{9}$$

with the tumor becoming increasingly necrotic.

Asymptotic tumor size

The final size achieved by a tumor in a host depends on the total resources the tumor can siphon from the host before killing it. If, on average, death occurs when a set, mass-independent fraction of host metabolic energy is appropriated by the tumor (or a set fraction of organ metabolic function is destroyed by an invading tumor), then the total tumor metabolic burden is $\sum_T B_T \propto B$, where the sum is over all tumors in the body at the time of death. In the simplified case where a single tumor dominates the tumor burden, this yields a prediction for the scaling of total tumor mass at death with host size: $M_T \propto M^{9/8}$.

Data Selection and Analysis

We culled the literature for available empirical data to test the many predictions of our theory. We provide references for these data, which also explain the methods used to collect those data. All datasets were fitted using the Matlab curve-fitting toolbox. The data in Figs. 2-3 were fitted using Type 1 linear regression. For tumor growth curves, nonlinear fits were made using a Trust-region algorithm. To estimate the size of the tumor growth transition from exponential to sigmoidal phases, a small sample of early-time growth data was fitted with an exponential, and late-time data points were fitted to the sigmoidal functional form. Remaining intermediate data were added to the exponential regime if they fell within the 95% confidence intervals for the initial exponential fit or were added to the sigmoidal phase if they fell within the 95% confidence intervals for the initial sigmoidal fit. Because a sigmoidal curve provides a reasonable description to the full range of data, empirical data that were within the 95% confidence intervals of both initial fits were included as part of the sigmoidal phase. After assigning the data according to this algorithm, new and final fits were calculated for the exponential and sigmoidal phases, and these fits were smoothly connected at their intersecting point. The coarseness of the data preclude the ability to rigorously determine the growth phases by statistics alone. Therefore the fits displayed are motivated strongly by our theory and should be considered demonstrative of our general approach. These fits often do not differ significantly from fitting the entire range of data with a sigmoidal curve, which represents a more phenomenological approach as in Guiot et al [2].

Data References

Data for figure 2 on tumor vascular branching properties taken from [3–5]. Data for figure 3 on the scaling of viable mass with total tumor size from [6]. Data for tumor growth curves in figure 4 from [7].

References

1. Sevick EM, Jain RK (1989) Geometric resistance to blood flow in solid tumors perfused ex vivo: Effects of tumor size and perfusion pressure. Cancer Res 49: 3506-3512.

2. Guiot C, Degiorgis P, Delsanto P, Gabriele P, Deisboeck T (2003) Does tumor growth follow a "universal law"? J Theor Biol 225: 147-151.

3. Less JR, Skalak TC, Sevick EM, Jain RK (1991) Microvascular architecture in a mammary carcinoma: branching patterns and vessel dimensions. Cancer Res 51: 265-273.

4. Less JR, Posner MC, Skalak TC, Wolmark N, Jain RK (1997) Geometric resistance and microvascular network architecture of human colorectal carcinoma. Microcirculation 4: 25-33.

5. Leunig M, Yuan F, Menger MD, Boucher Y, Goetz AE, et al. (1992) Angiogenesis, microvascular architecture, microhemodynamics, and interstitial fluid pressure during early growth of human adenocarcinoma ls174t in scid mice. Cancer Res 52.

6. Baker GM, Goddard HL, Clarke MB, Whimster WF (1990) Proportion of necrosis in transplanted murine adenocarcinoma and it's relationship to tumor growth. Growth, Development and Aging 54: 85-93.

7. Steel GG (1977) Growth kinetics of tumours. Clarendon Press.
